# Structural and Functional Analysis of the Engineered Type I DNA Methyltransferase EcoR124I_NT_

**DOI:** 10.1016/j.jmb.2010.03.008

**Published:** 2010-05-07

**Authors:** James E N Taylor, Phil Callow, Anna Swiderska, G. Geoff Kneale

**Affiliations:** 1 Biophysics Laboratories, Institute of Biomedical and Biomolecular Sciences, University of Portsmouth, Portsmouth PO1 2DT, UK; 2 Partnership for Structural Biology, Institut Laue Langevin, 38042 Grenoble Cedex 9, Grenoble, France

**Keywords:** DNA methyltransferase, DNA methylation, restriction–modification, sedimentation velocity, small-angle neutron scattering

## Abstract

The Type I R–M system EcoR124I is encoded by three genes. HsdM is responsible for modification (DNA methylation), HsdS for DNA sequence specificity and HsdR for restriction endonuclease activity. The trimeric methyltransferase (M_2_S) recognises the asymmetric sequence (GAAN_6_RTCG). An engineered R–M system, denoted EcoR124I_NT_, has two copies of the N-terminal domain of the HsdS subunit of EcoR124I, instead of a single S subunit with two domains, and recognises the symmetrical sequence GAAN_7_TTC. We investigate the methyltransferase activity of EcoR124I_NT_, characterise the enzyme and its subunits by analytical ultracentrifugation and obtain low-resolution structural models from small-angle neutron scattering experiments using contrast variation and selective deuteration of subunits.

## Introduction

Restriction–modification (R–M) enzymes provide a bacterial defence mechanism against foreign DNA. Hemi-methylated host DNA is fully methylated at specific sequences by a methyltransferase (MTase), thus protecting its DNA from restriction by the accompanying endonuclease (ENase). Foreign DNA is unmethylated at these sites and is cleaved.^1^, ^2^

Type I R–M systems are hetero-oligomeric enzymes encoded by three *hsd* (host specificity of DNA) genes encoding three polypeptides: HsdS, responsible for DNA recognition, HsdM for DNA modification and HsdR for cleavage. The ENase requires all three subunits: M, S and R while the MTase requires just the M and S subunits. For enzyme activity, the MTase is dependent upon *S*-adenosylmethionine, while the ENase in addition requires Mg^2+^ and ATP. All Type I R–M systems methylate a specific adenine at the N^6^ position (for reviews, see Ref. 3).

DNA sequence alignments of the S subunits of Type I R–M systems have shown the presence of two variable regions that form target recognition domains (TRDs), each recognising one-half of the bipartite DNA recognition motif, and two conserved regions that are believed to interact with the M subunit. The DNA recognition sequence of Type I R–M systems is, in general, asymmetric, and the two TRDs within the S subunit have different amino acid sequences. Typically, each of the DNA sequence half-sites is 3–5 bp in length, separated by a nonspecific spacer region (5–8 bp). On the basis of internal sequence homologies, a circular arrangement of the domains of the S subunit was suggested, which brings the N- and C-termini into close proximity.^4^ Circular permutations of the sequence of the N- and C-terminal conserved regions of the S subunit of EcoAI support this notion.^5^

Crystal structures have been reported for the Type I S subunits of *Mycoplasma genitalium* [Protein Data Bank (PDB) code: 1YDX] and *Methanococcus jannaschii* (PDB code: 1YF2), confirming the proximity of the N- and C-termini and in each case showing a hetero-dimeric structure held together by the interaction of coiled-coil regions.^6^, ^7^ Crystal structures are also available in the protein structure database for two M subunits—EcoKI (PDB code: 2AR0) and StySJI (PDB code: 2OKC), although both structures have significant regions of missing density (and neither has been published). More recently, structures of the R subunits of EcoR124I^8^ (PDB code: 2W00), *Vibrio vulnificus* YJ016^9^ **(**PDB code: 3H1T), and *Bacteroides fragalis* (PDB code: 3EVY; unpublished results) have been reported. No crystal structures have yet been reported for either a Type I MTase or an ENase, although various models have been proposed.^10^, ^11^, ^12^

EcoR124I is one of the best studied Type I R–M system; it recognises the asymmetric DNA sequence GAAN_6_RTCG.^13^, ^14^ Structural analysis of the full-length S subunit of EcoR124I has been hampered by the insolubility of this subunit unless co-expressed with the M subunit.^15^, ^16^ Various fragments of the *hsdS* gene were generated by PCR and over-expressed to allow further characterisation of the S subunit,^17^ and two of these proteins were found to be soluble. One of these (hereafter denoted S_NT_) corresponds to residues 1–215 of the parent S subunit and contains the N-terminal TRD and the central conserved region. This domain recognises the GAA of the parent recognition sequence and dimerises to recognise the symmetrical sequence GAAN_7_TTC.^18^, ^19^ As this system is based on the N-terminal domain of the specificity subunit of EcoR124I, it will be designated EcoR124I_NT_, although to date there has been no characterisation of its enzyme activity. There are clear similarities between M.EcoR124I_NT_ and M.AhdI, in which the S subunits each containing one TRD combine to form a homodimer, giving rise to a symmetrical recognition sequence.^20^, ^21^ Although the AhdI MTase has all the hallmarks of a Type I MTase, it should be noted that the AhdI ENase is quite unrelated to the AhdI MTase and, in this respect, resembles a Type II ENase.

Here, we investigate the methylation activity of the engineered MTase, M.EcoR124I_NT_. We also show that M.EcoR124I_NT_ is inhibited by ocr (“overcome classical restriction”), a small negatively charged protein that mimics DNA and whose biological role is to inhibit Type I R–M enzymes by competitive inhibition at the DNA binding site.^22^, ^23^ M.EcoR124I_NT_ and its component subunits have been further characterised by analytical ultracentrifugation. Finally, small-angle neutron scattering (SANS) experiments employing selective deuteration and contrast variation have allowed us to obtain low-resolution structural models of the MTase and to determine the spatial location of its subunits.

## Results and Discussion

### Methylation activity

**Fig. 1:**
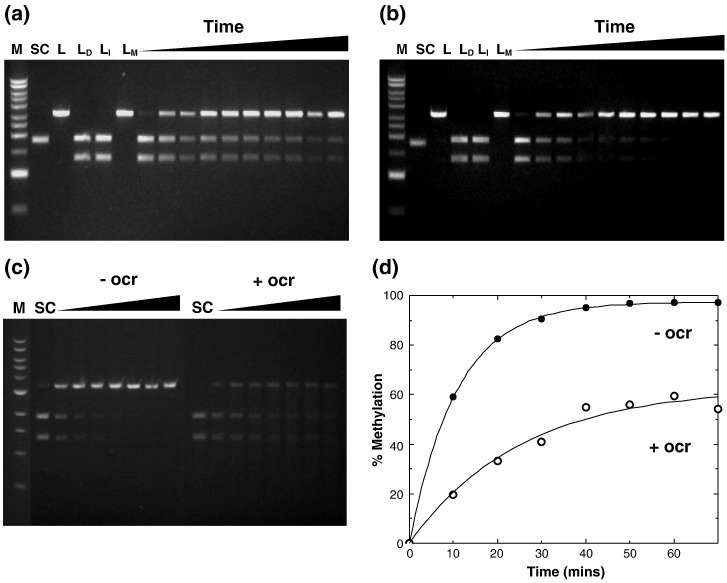
Methylation protection assays. (a) In the presence of 10 mM MgCl_2_. (b) In the absence of MgCl_2_. (c) MTase inhibition by ocr. In the presence and absence of an equimolar ratio of ocr in a buffer containing 10 mM MgCl_2_. (d) Enzyme progression curves for (c). Filled circles and open circles represent percent methylation in the absence and presence of ocr, respectively. All reactions were carried out at 37 °C and contained 2.5 nM DNA and 50 nM MTase [plus 50 nM ocr dimer, in the case of (c)]. M, 1 kb DNA Ladder (NEB or GE Healthcare); SC, supercoiled DNA; L, linear DNA, L_D_, linearised DNA followed by digestion with EcoRI; L_I_, inactivated M.EcoR124I_NT_ incubated with linearised DNA followed by EcoRI digestion; L_M_, linearised methylated DNA. Lanes beneath the triangles show aliquots taken at  10-min intervals and digested with EcoRI.

M.EcoR124I_NT_ was reconstituted from its individual subunits, and the complex was further purified as outlined in Materials and Methods. To assess the *in vitro* methylation activity of M.EcoR124I_NT_, we developed an assay, based on the prevention or otherwise of DNA cleavage by EcoRI. A plasmid was constructed in which the N_7_ spacer within the M.EcoR124I_NT_ recognition sequence (GAAN_7_TTC) was designed to contain a TTC sequence next to the 5′-GAA. This created an EcoRI recognition site (GAATTC), allowing the N^6^ methylation status of the second adenine to be monitored. In the absence of methylation, the  3127-bp linearised DNA substrate is digested by EcoRI into two DNA fragments of length 1834 bp and 1293 bp. These experiments showed that M.EcoR124I_NT_ had methylation activity that was independent of MgCl_2_ (Fig. 1a and b).

Inhibition by the ocr protein *in vitro* was then investigated. Previous studies have shown that ocr inhibits the DNA methylation, restriction, and ATPase activity of other Type I R–M systems such as EcoKI and EcoBI.^22^ The assay was conducted in the same way as the MTase assay, except that ocr was first incubated with M.EcoR124I_NT_ prior to DNA addition. It was found that at equimolar ratios of an ocr (dimer) to M.EcoR124I_NT_, there was approximately 50% MTase inhibition (Fig. 1c). Since ocr typically inhibits Type I MTases, in this respect, the engineered enzyme behaves similarly to other Type I enzymes.

### Hydrodynamic characterisation

In order to determine the molecular mass and stoichiometry in solution, we carried out sedimentation velocity (SV) and sedimentation equilibrium (SE) experiments on M.EcoR124I_NT_ and its constituent subunits. In each case, the sedimentation profiles were fitted with *c*(*s*) analysis using SEDFIT.^24^

**Fig. 2:**
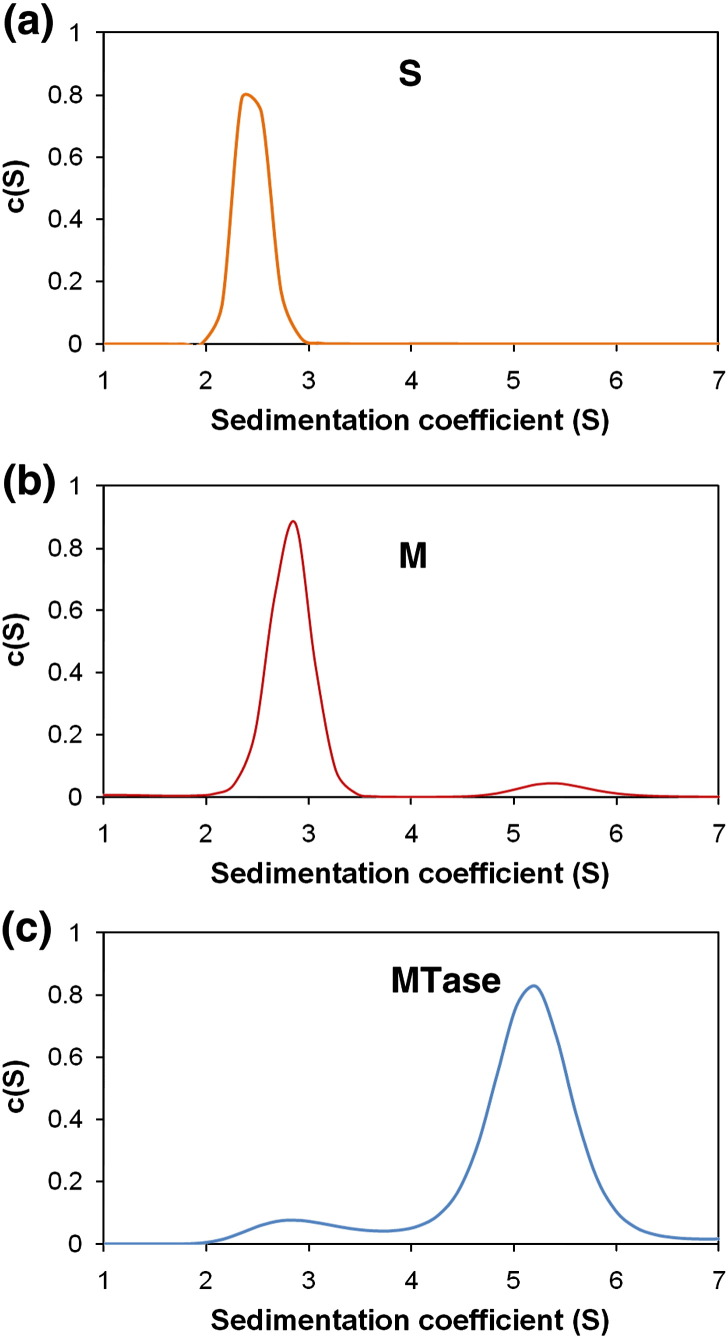
SV analysis of the MTase and its subunits. The *c*(*s*) distribution plots are shown for (a) S^NT^ at 25 μM, (b) M at 6 μM and (c) M.EcoR124I_NT_ at 2.6 μM. All experiments were performed at 30,000 rpm (in a Beckman Coulter An50 Ti analytical rotor), at 10 °C in buffer A, scanning every 12 min, at 280 nm.

SV experiments were first carried out on the S_NT_ and M subunits, which both sedimented essentially as a single species. Experimental sedimentation coefficients (*s*^⁎^) of 2.5 S and 2.9 S for S_NT_ and M, respectively, were obtained from the *c*(*s*) distribution profiles (Fig. 2a and b), which, when corrected, gave *s*_20__,w_ values of 3.3 S and 3.9 S, respectively. Following transformation to a *c*(*M*) distribution, experimental *M*_r_ values of 49,000 and 57,000 were obtained for S_NT_ and M, respectively, in excellent agreement with the theoretical *M*_r_ for a S_NT_ dimer (2 × 24,850) and an M monomer (58,000).

SV was then performed on M.EcoR124I_NT_ (Fig. 2c). Again, the sample was seen to exist almost entirely as a single species, with an *s*^⁎^ of 5.3 S and an *s*_20,w_ of 7.0 S, which are in reasonable agreement with values of 5.1 S and 6.7 S for the WT MTase (data not shown), suggesting that the two enzymes have a similar shape and structural organization. The *M*_r_ obtained from the *c*(*M*) distribution was 160,000, which is in close agreement with the expected value for a hetero-tetramer consisting of two S^NT^ and two M subunits (165,680). Table 1 summarises the hydrodynamic parameters that were obtained from SV.

**Table 1:** AUC parameters for M.EcoR124I_NT_ and its individual subunits

Species	*S*^a^	*s*_20__,w_^b^	*M*_r_^c^	*f*/*f*_o_^d^	*D*_20,w_^e^
S_NT_	2.5	3.3 (3.6)	49,000 (49,700)	1.48	6.2 (6.4)
M	2.9	3.9 (3.6)	57,000 (58,000)	1.39	6.1 (5.6)
MTase	5.3	7.0 (6.7)	160,000 (165,680)	1.55	3.9 (3.7)

*S* and *D* values predicted from the *ab initio* models using HYDROPRO are shown in parentheses (taking the S_NT_ dimer and the M monomer). Theoretical molecular weights for an S_NT_ dimer and an M monomer are also shown in parentheses.

a Experimental sedimentation coefficient (in Svedbergs).

b Corrected sedimentation coefficient (in Svedbergs).

c Experimental *M*_r_.

d Frictional ratio calculated assuming 0.4 g water per gram of protein.

e Experimental diffusion coefficient (× 10^− 7^ cm^2^ s^− 1^).

**Fig. 3:**
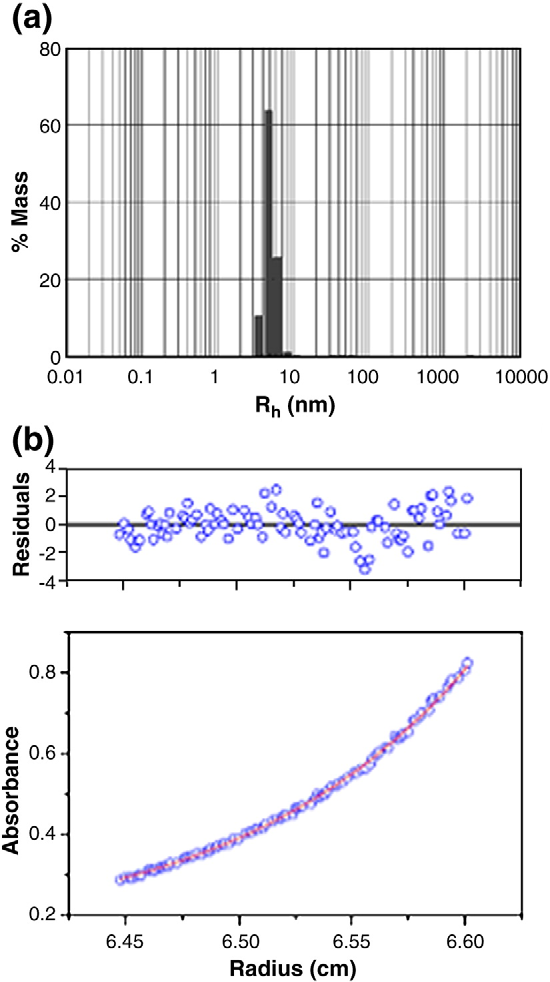
(a) DLS of M.EcoR124I_NT_ at a concentration of 5 μM at 10 °C. A hydrodynamic radius of 5 nm with a polydispersity of 0.87 nm (7.4%) was obtained. (b) SE of M.EcoR124I_NT_. Shown is an example fit from data collected at 8500 rpm (using a Beckman Coulter An50 Ti analytical rotor), scanned at 276 nm (after 21 h), at a concentration of 5 μM at 10 °C. The data were fitted to a single ideal species model and yielded an apparent *M*_r_ of 162,100. The top panel shows the residual error between the fitted and experimental values.

SE was conducted within a concentration range of 2.5 to 8.8 μM to obtain a more accurate value for the molecular mass in solution of the MTase. The data fitted well to a single ideal species model, as noted from the random distribution of the residuals about zero (a representative fit is shown in Fig. 3b). The experimental *M*_r_ was 163,000, using a global fit from runs carried out at 6500 rpm and 8500 rpm, again in good agreement with the theoretical *M*_r_ for a hetero-tetramer of 165,680.

### Small-angle neutron scattering

Dynamic light scattering (DLS) was routinely used to check for mono-dispersity of the MTase, where a single peak with a hydrodynamic radius of approximately 4.5–5.0 nm was typically found (Fig. 3a). Having confirmed that the M.EcoR124I_NT_ complex was mono-disperse and with no tendency to aggregate, we were able to carry out SANS experiments. SANS allows the low-resolution structure of macromolecular complexes to be determined. If specific subunits can be deuterated, then contrast variation can be used to allow the location of the subunits to be established. Unlike the WT enzyme, M.EcoR124I_NT_ can be reconstituted from its individual subunits, thus allowing the deuteration of specific subunits, which can then be matched out in buffers containing D_2_O.

**Fig. 4:**
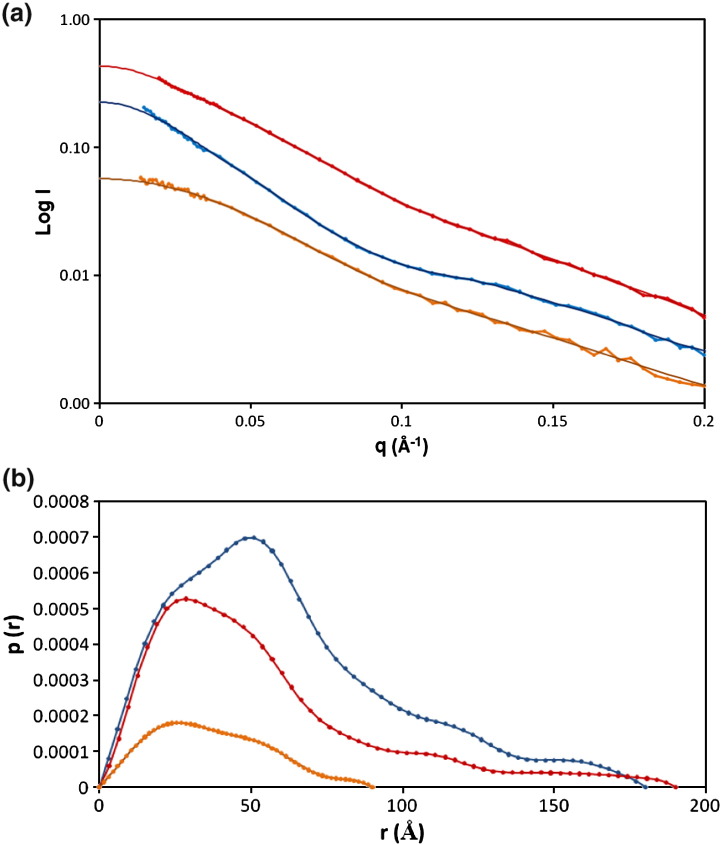
Small-angle neutron scattering. (a) SANS scattering curves for M.EcoR124I_NT_ measured in 100% D_2_O (blue), M.EcoR124I_NT_ containing deuterated S_NT_ measured in 40% D_2_O (orange) and M.EcoR124I_NT_ containing deuterated S_NT_ measured in 100% D_2_O (red). In each case, the continuous curve is the corresponding fit from GNOM. (b) Distance distribution functions obtained by transformation of the data shown in (a).

Scattering curves were first collected for the fully hydrogenated M.EcoR124I_NT_ in 100% D_2_O (Fig. 4a). Following the construction of Guinier plots, a radius of gyration (*R*_g_) of 52.9 Å was obtained. The scattering curve was subsequently transformed to a distance distribution function, *p*(*r*), and the maximum dimension, *D*_max_ [i.e., when *p*(*r*) = 0], was estimated to be 180 Å for the MTase (Fig. 4b).

Scattering data were then measured for a complex of M.EcoR124I_NT_ composed of deuterated S_NT_ and hydrogenated M subunits. In 40% D_2_O, we obtained an *R*_g_ of 32.4 Å and a *D*_max_ of 90 Å; these values represent the structure of the S_NT_ subunits in the complex, since the M subunits are matched out. For the same sample in 100% D_2_O, values of *R*_g_ = 50.1 Å and *D*_max_ = 190 Å were obtained; these values correspond to the structure of the two M subunits in the complex, as the deuterated S_NT_ subunits are now matched out. Our results (Table 2) clearly indicate that the M subunits extend towards the periphery of the complex, whereas the S subunits are located more centrally.

**Table 2:** SANS parameters for M.EcoR124I_NT_ and its subunits *in situ*

	% D_2_O	*R*_g_ (Å)	*D*_max_ (Å)
MTase^a^	100	52.9	180
S_NT_ subunits^b^	40	32.4	90
M subunits^c^	100	50.1	190

a M.EcoR124I_NT_ measured in 100% D_2_O.

b M.EcoR124I_NT_ with deuterated S_NT_ subunits measured in 40% D_2_O.

c M.EcoR124I_NT_ with deuterated S_NT_ subunits measured in 100% D_2_O.

Since X-ray crystal structures are available for homologues of each of the subunits of M.EcoR124I_NT_, it is instructive to compare the predicted and experimental SANS parameters for the subunits within the MTase. Using HYDROPRO to calculate *R*_g_ and *D*_max_ from the crystal structure of the HsdS dimer of *M. jannaschii* (PDB code: 1YF2), we obtain values of 30.5 Å and 91 Å, respectively, which compare very well with the experimental values from SANS (32.5 Å and 90 Å). Likewise, the predicted scattering curve from the crystal structure fits the data extremely well up to *Q* = 0.14 Å^− 1^ (see Supplementary Fig. S1a). In contrast, the predicted *R*_g_ and *D*_max_ for the EcoKI HsdM dimer (40.5 Å and 157 Å, respectively) differ significantly from the experimental SANS parameters (50.1 Å and 190 Å) and the predicted scattering curve is not remotely similar to the experimental curve (see Supplementary Fig. S1b). The poor correspondence between the EcoKI M dimer in the crystal and the experimental SANS data for M.EcoR124I_NT_ is not unexpected as (1) the sequence homology between the M subunits of EcoR124I and EcoKI is not strong, (2) a significant fraction of the electron density map of the EcoKI M subunit is missing and (3) the EcoKI dimer of M subunits in the unit cell may not have the same interactions in the MTase—in fact, for EcoR124I_NT_, we show that the M subunit exists as a monomer in solution.

**Fig. 5:**
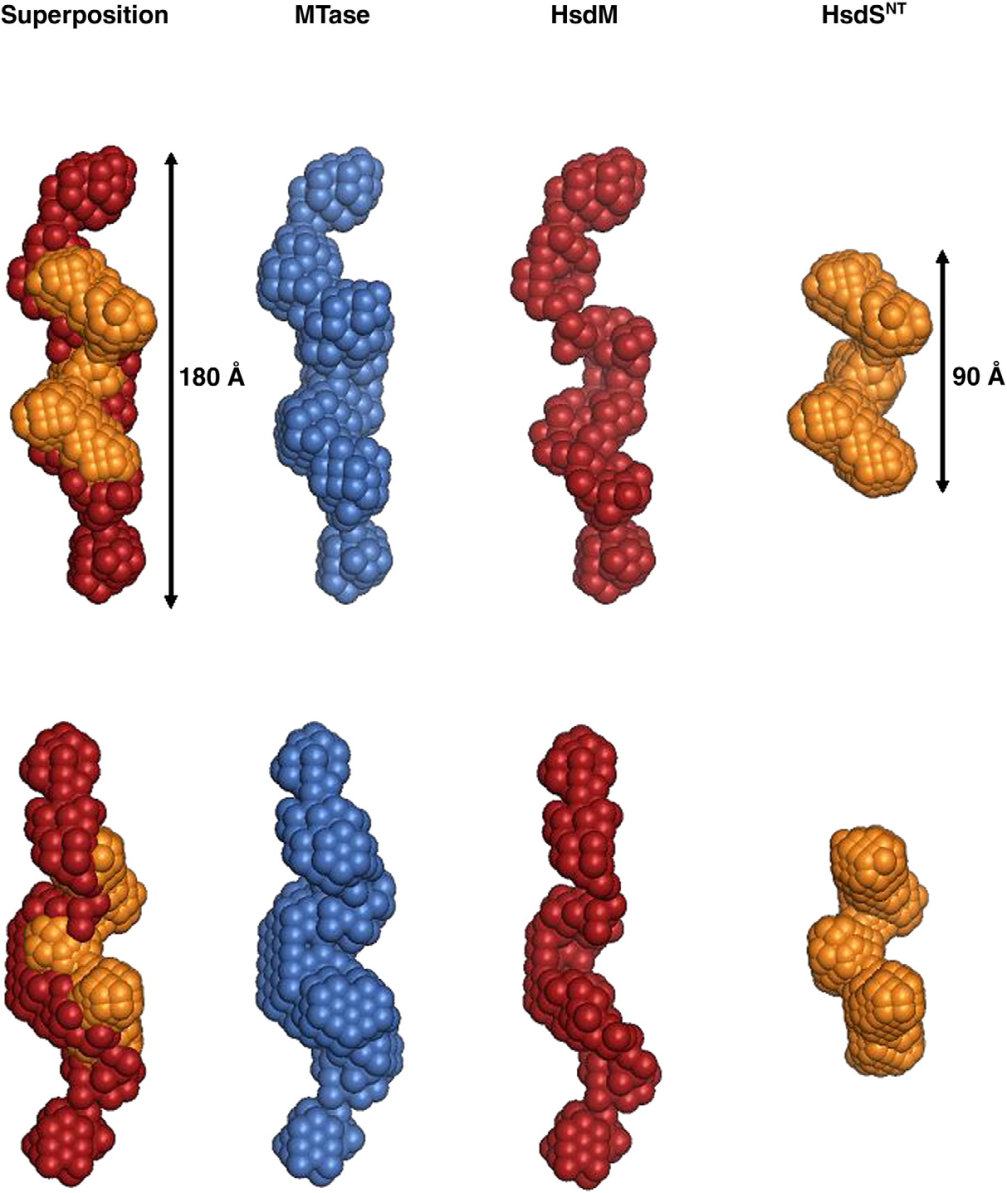
*Ab initio* models of M.EcoR124I_NT_ (blue), and the M and S_NT_ subunits *in situ* (red and orange, respectively). Twenty DAMMIN models were averaged using DAMAVER and models were aligned using SUPCOMB. In all cases, *P*2 symmetry was imposed. The two rows show mutually perpendicular views of the structures, rotated by 90° around the *y*-axis.

Low-resolution *ab initio* models were then constructed using DAMMIN to model the SANS data (Fig. 5). Typically, for each experiment, 20 independent runs of DAMMIN were performed, and the resulting models were filtered and averaged by DAMAVER (see Materials and Methods). This process was repeated a number of times, and the final *ab initio* models obtained in each case were found to have the same general features.

The *ab initio* model for the shape of M.EcoR124I_NT_ was determined by analysis of the data from the fully hydrogenated enzyme measured in D_2_O buffer. The *ab initio* model for the S subunits was determined by analysis of scattering data obtained from the MTase reconstituted with deuterated S_NT_ subunits and measured in 40% D_2_O buffer. The *ab initio* shape determined for S_NT_ reveals that the two subunits dimerise into a typical Z-shaped structure resembling the S subunits of both *M. genitalium* and *M. jannaschii*.^6^, ^7^ The model of the M subunits was obtained by subtracting the model of the S subunits from that of M.EcoR124I_NT_.

The program HYDROPRO was then used to calculate hydrodynamic parameters for each of the *ab initio* models. Table 1 compares the hydrodynamic parameters from AUC with those predicted from the *ab initio* models of the MTase and its subunits. Considering the low resolution of the bead models, the agreement between predicted and experimental values of sedimentation coefficient and diffusion coefficient is very good (within 3–9%). This provides further evidence to support the *ab initio* model of the EcoR124I_NT_ MTase and indicates that the subunits do not undergo any large-scale structural changes on forming the MTase.

We attempted to fit crystal structures of homologous subunits (the M dimer of EcoKI and the S subunit of *M. jannaschii*) to the *ab initio* models of the M and S components of EcoR124I_NT_. As expected, the fit to the S subunit model was good, but the fit to the M subunit model was less so (see Supplementary Fig. S2) and a unique orientation could not be defined with any certainty for the latter, even if the location and the orientation of the individual M subunits were allowed to vary. The resolution of the technique was not considered adequate to fit the M and S_NT_ subunits simultaneously to the *ab initio* model of M.EcoR124I_NT_ as there are numerous ways of fitting these subunits together.

The fit between the *ab initio* model of the S_NT_ dimer and the crystal structure shows that the two half subunits of the latter dimerise to form a similar structure to that of an intact S subunit, which appears to be well conserved at the structural level between unrelated R–M systems. In contrast, the EcoKI dimer, as found in the crystal structure, does not match the M subunit organisation within M.EcoR124I_NT_. The results of the SANS analysis suggest that the M subunits are linked essentially end to end in the MTase, making at most rather limited contacts at the centre of the complex. Even though the free M subunits exist as monomers in solution, there may be relatively weak protein–protein contacts between them in the MTase, presumably stabilised by interactions with the S subunits, which form a very stable dimer.

The overall low-resolution structure we have obtained for M.EcoR124I_NT_ resembles that of the MTase, M.AhdI, as determined by SANS.^21^ The interactions of the M subunits in the *ab initio* model are rather different to those proposed for M.EcoKI on the basis of electron microscopy^10^ or for M.EcoR124I on the basis of molecular modelling.^11^ However, it should be noted that those models were for complexes formed with DNA (or the DNA mimic, ocr) and such structures are likely to be much more compact than the free protein, which exists in the “open” conformation.^25^

## Materials and Methods

### Protein Purification

The expression and purification of S_NT_ were carried out as previously published.^17^ For the M subunit, a  5-mL starter culture was grown until OD_600_ (optical density at 600 nm) reached 0.6 and was used to inoculate flasks containing 500 mL 2 × YT, which were also grown to OD_600_ of 0.6. The M subunit was expressed overnight following induction with 1 mM isopropyl-β-d-thiogalactopyranoside. The cells were harvested by centrifugation at 39,000 rpm at 4 °C for 30 min. The cell pellets were stored at − 20 °C. The pellet was resuspended at 4 °C in 50 mM Tris–HCl, pH 8.0, 25% w/v sucrose, and 1 mM Na_2_EDTA (disodium ethylenediaminetetraacetic acid), followed by sonication and centrifugation at 39,000***g*** for 30 min at 4 °C. The clarified lysate was supplemented with protamine sulphate (Sigma) to a final concentration of 20 mg/mL and 500 mM NaCl, mixed slowly at 4 °C for 30 min and then centrifuged at 39,000***g*** for 20 min at 4°C.

The M subunit was finally purified using a HiTrap™ desalting (GE Healthcare) column equilibrated in buffer A (10 mM Tris–HCl, pH 8.0, 100 mM NaCl, and 1 mM Na_2_EDTA). This step produces pure M subunit since it unexpectedly (but reproducibly) binds to the column during buffer exchange and elutes with the leading edge of the salt peak; in contrast, the contaminating proteins elute, as expected, in the void volume. The M subunit was subsequently dialysed into buffer A and remained soluble and mono-disperse as judged by analytical ultracentrifugation.

The multisubunit M.EcoR124I_NT_ enzyme was formed by incubation of purified S_NT_ and M subunits for 30 min at 4 °C. The sample was applied to a  5-mL HiTrap™ heparin column (GE Healthcare) equilibrated in buffer A, and a linear gradient of NaCl (0.1 M to 2.0 M) was applied at 1 mL/min over 10 column volumes. The intact M.EcoR124I_NT_ eluted at approximately 250 mM NaCl.

### Methylation and inhibition assays

A  30-bp DNA duplex incorporating the sequence GAATTCN_4_TTC (which includes the recognition sites for both M.EcoR124I_NT_ and EcoRI ) was blunt-end ligated into the SmaI site of the plasmid pUC119 EcoRI^−^ (which lacked any EcoRI sites—C. Dutta, personal communication) to form the plasmid pUC119/EcoR124I_NT_. The orientation, correct number of inserts and the lack of mutations were confirmed by DNA sequencing around the inserted sequence. Following linearization of this plasmid with XmnI, we incubated the samples with M.EcoR124I_NT_ at 37 °C. Fifteen-microliter aliquots were removed at various times and heat inactivated at 65 °C for 20 min. After cooling on ice for 10 min, each 15-μL sample was challenged with EcoRI and incubated for a further 60 min at 37 °C. The products of the reaction were run on a 0.8% agarose gel. The ocr inhibition assay was carried out in the same way, except that ocr was added at the appropriate molar ratio to M.EcoR124I_NT_ prior to the addition of DNA. Agarose gels were digitally photographed using a FujiFilm FLA-5000 phosphorimager and quantified using Image Gauge.

### Sedimentation velocity

SV experiments were carried out in a Beckman Optima XL-A analytical ultracentrifuge (Beckman Coulter, Brea, CA).  Four hundred microliters of sample (either S_NT_, M or M.EcoR124I_NT_) and 425 μL of buffer A were loaded into the corresponding sectors of a double-sector cell of 12 mm optical path length. The cells were loaded into an An50 Ti analytical rotor, which had been left overnight at 4 °C and transferred to the centrifuge, where it was left to equilibrate. The rotor was accelerated to 30,000 rpm, and readings of absorbance *versus* radial distance were taken every 12 min at 280 nm at 10 °C. The raw data were analysed using the program SEDFIT,^24^ using radial data within the range 6.06–7.00 cm. Partial specific volumes and buffer densities were calculated using the program SEDNTERP and corrected for temperature.^26^ The experimental sedimentation coefficients obtained from the *c*(*s*) distribution plot were finally corrected for temperature and solvent using SEDNTERP so that a *s*_20__,w_ value could be obtained.

### Sedimentation equilibrium

SE was carried out in a Beckman Optima XL-A analytical ultracentrifuge. Experiments were performed in six-channel cells of 12 mm optical path length, using 90 μL of sample (M.EcoR124I_NT_) at a protein concentration range from 2.5 to 8.8 μM.  One hundred microliters of buffer was loaded into the corresponding control channel. The cells were loaded into an An50 Ti analytical rotor at 4°C. The rotor was accelerated to 6500 rpm and 8,500 rpm, and scans of absorbance *versus* radial displacement were measured at a wavelength of 276 nm, at a resolution of 0.001 cm at 0, 15, 18 and 21 h. Finally, a meniscus depletion was carried out at 40,000 rpm.

### Dynamic light scattering

DLS was performed with purified MTase at 5 mM, at 10°C in buffer A, using a Protein Solutions DynaPro MSTC800 light-scattering instrument. The results from 30 measurements were averaged, and values for the hydrodynamic radius, *R*_h_, and polydispersity were obtained. The experimental molecular mass, *M*_r_, was estimated using the standard molecular weight model (Dynamics V5, Protein Solutions).

### Small-angle neutron scattering

#### Sample preparation

The S_NT_ subunit was deuterated by expression of the S_NT_ gene from pET-21a in BL21 (DE3) cells. Enfors minimal medium containing 85% D_2_O with hydrogenated glycerol as the carbon source was used to give a 75% deuteration level, such that the protein had a contrast match point equivalent to 100% D_2_O. The M.EcoR124I_NT_ complex was formed either as the fully hydrogenated enzyme or as a partially deuterated complex by mixing the appropriate subunits, that is, with both S_NT_ and M hydrogenated, or with deuterated S_NT_ and hydrogenated M. The complex was purified as described above. Complexes were then dialysed into buffer A in varying H_2_O/D_2_O ratios.

#### SANS measurements

Data were collected using the D22 diffractometer at the ILL using two detector distances, 2 m and 8 m, covering a *Q* range of 0.008–0.35 Å^− 1^ at a wavelength of 6 Å, where *Q* is the scattering vector (4πsinθ/λ). Scattering data were collected from a 96 cm × 96 cm detector with a pixel size of 7.5 mm × 7.5 mm. Data reduction was performed using the GRASansP software (Dewhurst, 2006†). Modelling of the SANS data was performed using the ATSAS software package.^27^ Data from both distances were merged over the range 0.013 to 0.2 Å^− 1^ and evaluated using PRIMUS.^28^ At low angle, the scattering intensities *I*(*Q*) can be described by the Guinier approximation, *I*(*Q*) = *I*(0) exp 1/3 *R*_g_^2^*Q*^2^, where *R*_g_ is the radius if gyration. The isotropic scattering intensity *I*(*Q*) was transformed to the particle distance distribution function, *p*(*r*), using the program GNOM,^29^ which was used to estimate the particle maximum dimensions *D*_max_. Scattering curves were then generated by back transformation of each of these *p*(*r*) functions and compared to the experimental data. The value of *D*_max_ was confirmed when the *R*_g_ obtained from the *p*(*r*) distribution was equal to that obtained from the Guinier plot.

#### Ab initio modelling

Once the *p*(*r*) curves had been obtained for S^NT^, M and the MTase, DAMMIN was used to create low-resolution *ab initio* models.^27^ In all cases, *P*2 symmetry was imposed. The packing radii of the dummy atoms used for the modelling of the MTase and the M and S_NT_ subunits were 4.6 Å, 4.4 Å and 2.2 Å, respectively. Penalty weights for the looseness and disconnectivity were set to 3 × 10^− 3^ and a peripheral penalty weight of 0.3 was used. The final root-mean-square errors (chi) for all models were between 1.0 and 1.5. Typically, 20 models were aligned, averaged and filtered using DAMAVER, discarding any models that had a normalized spatial distribution higher than that of the mean plus twice the variation.^30^ The number of dummy atoms in the averaged models for the MTase, the M subunits and the S_NT_ subunits were 482, 661 and 394, respectively. *Ab initio* models were overlaid using the program SUPCOMB, taking the MTase *ab initio* model as a template.^31^ All models were visualized using the program PyMOL.

Hydrodynamic parameters for each model were calculated using the program HYDROPRO.^32^ Small-angle scattering curves were generated from atomic resolution models using the program CRYSON.^33^

## Appendix A

Supplementary Fig. S1. Simulated scattering data from atomic structures (blue curves) *versus* experimental SANS data (red circles). (a) M.EcoR124I_NT_ containing deuterated S_NT_ measured in 40% D_2_O together with the simulated scattering data from the crystal structure of the HsdS subunit of *M. jannaschii* (PDB code: 1YF2). (b) M.EcoR124I_NT_ containing deuterated S_NT_ measured in 100% D_2_O, together with the simulated scattering data from the crystal structure of the HsdM dimer of EcoKI (PDB code: 2AR0).

Supplementary Fig. S2. Overlays of related crystal structures (represented as ribbons) onto the *ab initio* models of the subunits of M.EcoR124I_NT_. (a) Two copies of the M subunit, taken from the EcoKI crystal structure (PBD code: 2AR0) superimposed on the *ab initio* model of the HsdM dimer. (b) Crystal structure of the S subunit of *M. jannaschii* (PBD code: 1YF2) superimposed on the *ab initio* model of the S_NT_ dimer.

## Footnotes

† www.ill.fr/lss/grasp/grasp_main.html
