# Supplementary material for: Structural and Functional Analysis of the Engineered Type I DNA Methyltransferase EcoR124INT
Source: J Mol Biol. 2010 May 7;398(1):391–9. doi: 10.1016/j.jmb.2010.03.008 (PMC2877798; doi:10.1016/j.jmb.2010.03.008)
Supplement: Supplementary Fig. S1. — Simulated scattering data from atomic structures (blue curves) versus experimental SANS data (red circles). (a) M.EcoR124INT containing deuterated SNT measured in 40% D2O together with the simulated scattering data from the crystal structure of the HsdS subunit of M. jannaschii (PDB code: 1YF2). (b) M.EcoR124INT containing deuterated SNT measured in 100% D2O, together with the simulated scattering data from the crystal structure of the HsdM dimer of EcoKI (PDB code: 2AR0). [file mmc1.doc]

(a)


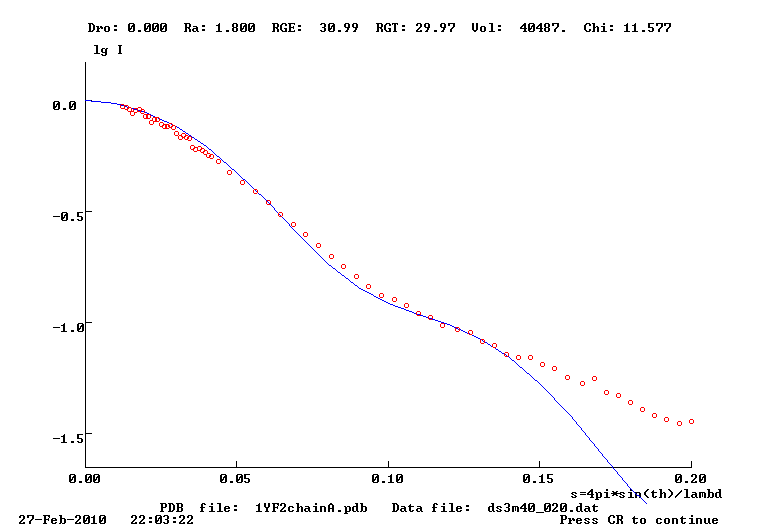

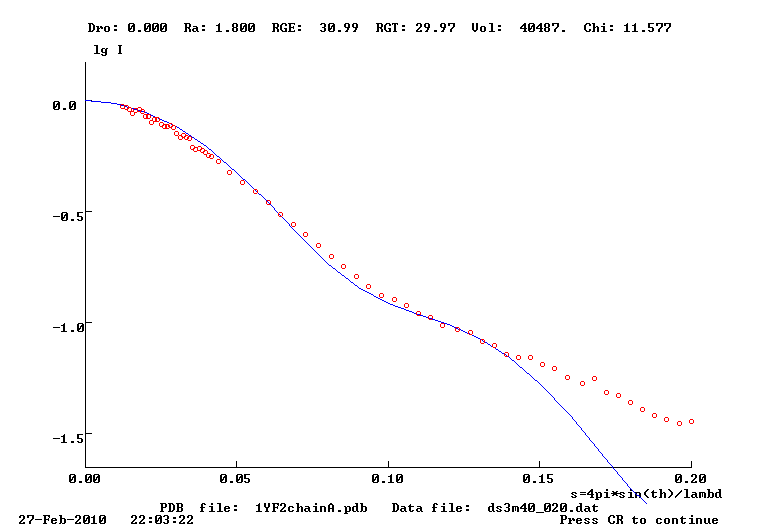


(b)


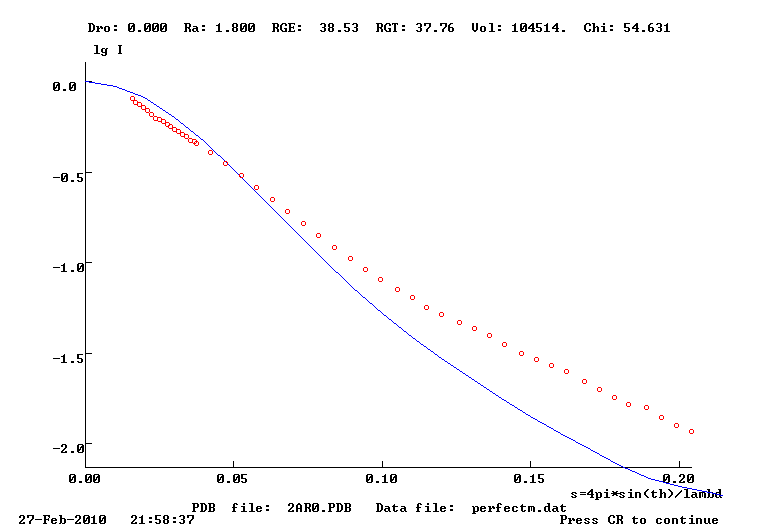

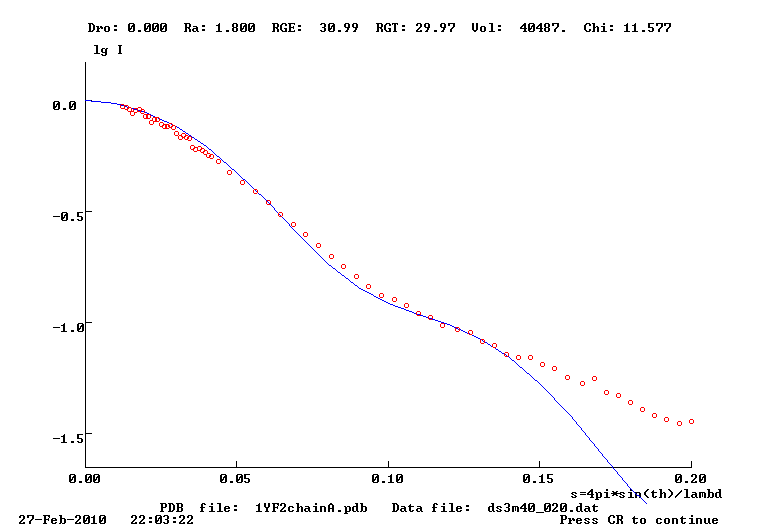


**Figure S1.** Simulated scattering data from atomic structures (blue curves) *versus* experimental SANS data (red circles). (a) M.EcoR124I_NT_ containing deuterated S_NT_ measured in 40 %D_2_O together with the simulated scattering data from the crystal structure of the HsdS subunit of *Methanoccusjannaschii*(PDB 1YF2). (b) M.EcoR124I_NT_ containing deuterated S_NT_ measured in 100 % D_2_O , together with the simulated scattering data from the crystal structure of the HsdM dimer of EcoKI (PDB 2AR0).
