# Supplementary material for: Structural and Functional Analysis of the Engineered Type I DNA Methyltransferase EcoR124INT
Source: J Mol Biol. 2010 May 7;398(1):391–9. doi: 10.1016/j.jmb.2010.03.008 (PMC2877798; doi:10.1016/j.jmb.2010.03.008)
Supplement: Supplementary Fig. S2. — Overlays of related crystal structures (represented as ribbons) onto the ab initio models of the subunits of M.EcoR124INT. (a) Two copies of the M subunit, taken from the EcoKI crystal structure (PBD code: 2AR0) superimposed on the ab initio model of the HsdM dimer. (b) Crystal structure of the S subunit of M. jannaschii (PBD code: 1YF2) superimposed on the ab initio model of the SNT dimer. [file mmc2.doc]

**(a)**

**(b)**


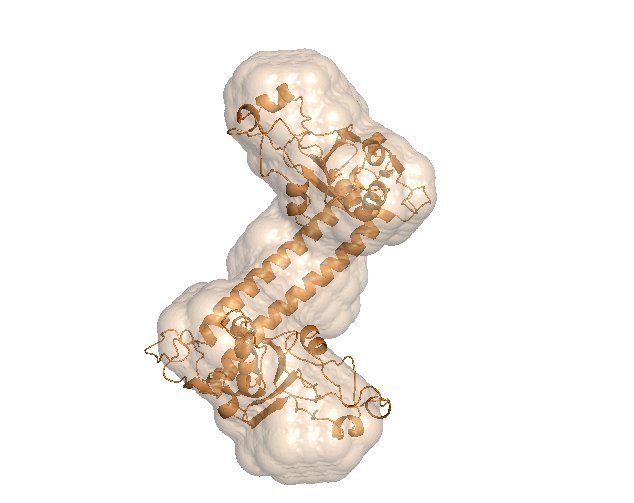

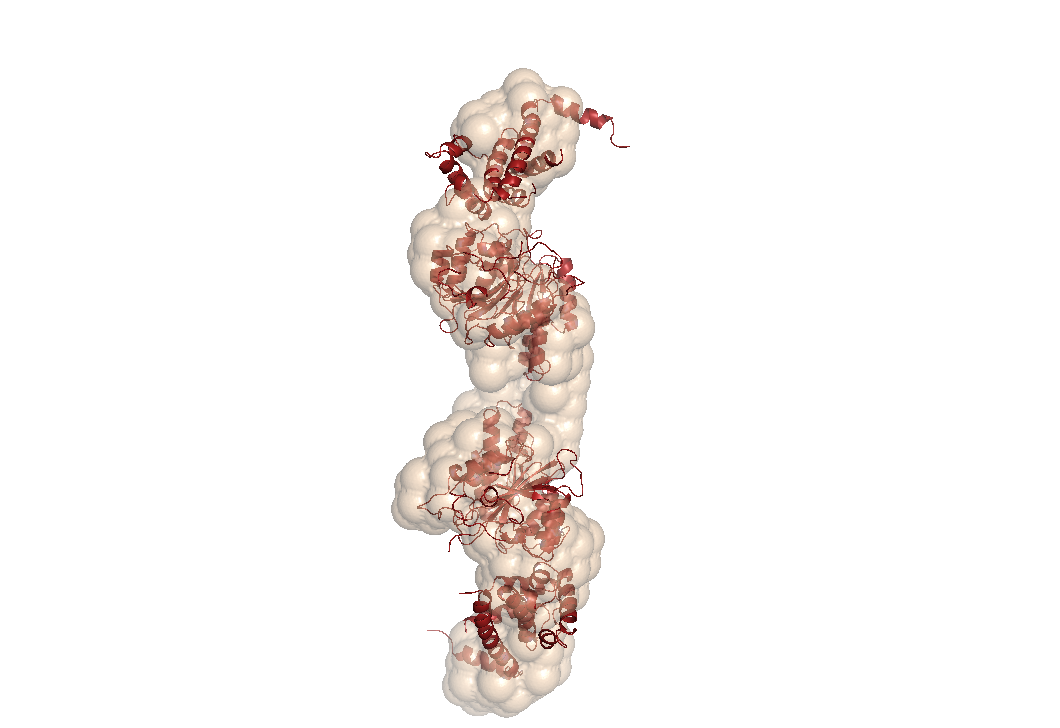


**HsdM subunit HsdS subunit**

Fig. S2. Overlays of related crystal structures (represented as ribbons) onto the *ab initio* models of the subunits of M.EcoR124I_NT_. (a) Two copies of the M-subunit, taken from the EcoKI crystal structure (PBD code 2AR0) superimposed on the *ab intio* model of the HsdM dimer. (b) crystal structure of the S subunit of [*Methanococcus jannaschii*](http://pdbbeta.rcsb.org/pdb/search/smartSubquery.do?smartSearchSubtype=TreeEntityQuery&t=1&n=243232)  (PBD code 1YF2) superimposed on the the *ab initio* model of the S_NT_ dimer.
